# Supplementary material for: Nucleoside Phosphorylases make N7-xanthosine
Source: Nat Commun. 2024 Apr 29;15:3625. doi: 10.1038/s41467-024-47287-4 (PMC11058261; doi:10.1038/s41467-024-47287-4)
Supplement: Supplementary file 1 — Supplementary Information [file 41467_2024_47287_MOESM1_ESM.pdf]

Nucleoside Phosphorylases Make *N*7-Xanthosine

Supplementary Information

Sarah Westarp,[1,2] Felix Brandt,[3] Lena Neumair,[1] Christina Betz,[1] Amin Dagane,[1] Sebastian Kemper,[4] Christoph R. Jacob,[3] Peter Neubauer,[1] Anke Kurreck,[1,2,§] and Felix Kaspar,[5,§]

[1] Chair of Bioprocess Engineering, Institute of Biotechnology, Faculty III Process Sciences, Technische Universität Berlin, Straße des 17. Juni 135, 10623, Berlin, Germany
[2] BioNukleo GmbH, Ackerstraße 76, 13355 Berlin, Germany
[3] Institute of Physical and Theoretical Chemistry, Technische Universität Braunschweig, Gaußstraße 17, 38106 Braunschweig, Germany
[4] Institute for Chemistry, Technische Universität Berlin, Straße des 17. Juni 135, 10623 Berlin, Germany
[5] Institute for Biochemistry, Biotechnology and Bioinformatics, Technische Universität Braunschweig, Spielmannstraße 7, 38106, Braunschweig, Germany
[§] anke.wagner@tu-berlin.de, felix.kaspar@web.de

Table of Contents

Supplementary Methods

General remarks 3
Enzymes 4
Transformations 6
Protein production and purification 6
Discovery of **N7X** (Figs. 1c and 1d) 8
HPLC analyses (Fig. 1e) 8
Activity screening of *Gt*PNP for production of **N7X** 9
Computational prediction of UV absorption spectra (Fig. 1f) 10
Purification of **N7X** and NMR analyses (Fig. 1g) 11
Structure elucidation of **N7X** 11
Reference UV spectra of **X** and **N7X** at pH 10 (Fig. 1h) 15
Absorption spectra of **X**, **N9X** and **N7X** as functions of pH (Fig. 1i) 16
Equilibrium constant of phosphorolysis of **N7X** (Figs. 1j and 1k) 17
Michaelis-Menten kinetics with *Gt*PNP (Fig. 1l) 18
Synthesis of **N7X**/**N9X** by various nucleoside phosphorylases (Fig. 1m) 19
Stability of **N7X** 21

Additional control experiments 22
NMR spectrum of **N7X** 23
Supplementary References 24

General remarks

All chemicals used in this study were of analytical grade or higher and purchased from Sigma Aldrich (Steinheim, Germany), Carl Roth (Karlsruhe, Germany), TCI Deutschland (Eschborn, Germany) or VWR (Darmstadt, Germany) and used without prior purification. Water deionized with a Sartorius or Werner water purification system was used for the preparation of all enzymatic reactions as well as for purification and storage buffers. Enzymatic reactions were generally prepared from stock solutions of substrate(s), salt(s), buffer(s) and enzyme(s) and started *via* the addition of enzyme or key reagent as indicated for the respective experiment. Stock solutions of xanthine (**X**) were prepared at 1 mM by sonication and addition of NaOH as needed (the sodium salt of **X** is much more soluble than the neutral species). UV absorption spectra and UV traces were recorded in acrylic microcuvettes (Sarstedt, Nümbrecht, Germany) with a Cary 60 UV-Vis Spectrometer (Agilent Technologies, Santa Clara, USA) or in UV-transparent 96 well plates (UV-Star®, Greiner Bio-One, Frickenhausen, Germany) with a CLARIOstar 96-well microplate reader (BMG Labtech, Ortenberg, Germany). Nuclear magnetic resonance (NMR) spectra were recorded on a Bruker AVIII 700 with the deuterated solvent acting as an internal deuterium lock. ^{1}H and ^{13}C NMR spectra are referenced to sodium trimethylsilylpropionate (TSP) as internal standard (0 ppm) or to the residual solvent signal. ^{31}P NMR chemical shifts are stated relative to phosphoric acid in water. Data are reported as follows: chemical shift (ppm), multiplicity (s = singlet, d = doublet, t = triplet, q = quartet, m = multiplet), coupling constant(s) (Hz), and integration. Data handling and routine calculations were carried out in Excel or LibreOffice, NMR data analysis in MestreNova (version 14.3), and analysis, fitting and data plotting in OriginPro (2022 version).

Enzymes

Geobacillus thermoglucosidasius purine nucleoside phosphorylase (*Gt*PNP, DeoD, WP_ 013400686.1), cloned from genomic DNA as described by Zhou *et al.*[1]

ε_{280} = 26025 M^{-1} cm^{-1}, E1% = 9.4

MMRGSHHHHHHGSLSIHIEAKQQEIAEKILLPGDPLRAQYIAETFLEGATCYNRVRGMLGFTGTYKGHRISVQGTGMG
VPSISIYVNELIQSYHVQTLIRVGTCGAIQKDVNVRDVILAMSASTDSNMNRLTFRGRDYAPTANFALLRTAYEVGAEK
GLPLKVGSVFTADMFYNDEPDWETWARYGVLAVEMETAALYTLAAKFGRKALSVLTVSDHILTGEETTAQERQTTFN
DMIEVALETAIRVE

Thermus thermophilus purine nucleoside phosphorylase I (*Tt*PNP or *Tt*PNP I, gene AA2Tth_RS07555, WP_011173486.1), cloned via BamHI/HindIII sites from gene synthesis (GeneArt, FisherScientific)

ε_{280} = 33920 M^{-1} cm^{-1}, E1% = 12.6

MMRGSHHHHHHGSSPIHVRAHPGDVAERVLLPGDPGRAEWIAKTFLQNPRRYNDHRGLWGYTGLYKGVPVSVQTT
GMGTPSAAIVVEELVRLGARVLVRVGTAGAASSDLAPGELIVAQGAVPLDGTTRQYLEGRPYAPVPDPEVFRALWRR
AEALGYPHRVGLVASEDAFYATTPEEARAWARYGVLAFEMEASALFLLGRMRGVRTGAILAVSNRIGDPELAPPEVL
QEGVRRMVEVALEAVLEV

Thermus thermophilus purine nucleoside phosphorylase II (*Tt*PNP II, gene WP_011172648.1), cloned via BamHI/HindIII sites from gene synthesis (GeneArt, FisherScientific)

ε_{280} = 21890 M^{-1} cm^{-1}, E1% = 6.8

MMRGSHHHHHHGSPDMELYDKIQEAVAYVRSKTDFVPEVGLVLGSGLGPLADEVEKVAEIPYGEIPHFPVSTAPGHA
GRLVLGRLEGKPVLVYKGRVHYYEGYSAEEVVFPVRVGFFLGARTFLLTSAAGGLNPRFRAGGIMLHLDYINFAGANP
LRGPNDERLGPRFPVMFEAYDPELIELARKVARRQDLHLFEGVYAWFMGPSFASRAELRLLRELGADAIGMSTVPEVI
ALRHLGARVLGLSTITDMAVPEREHHATEEEVLRVAAETGPVFRRYVRGILAEL

Escherichia coli purine nucleoside phosphorylase (*Ec*PNP, WP_000224877.1) cloned via BamHI/HindIII sites from gene synthesis (GeneArt, FisherScientific)

ε_{280} = 8940 M^{-1} cm^{-1}, E1% = 3.2

MMRGSHHHHHHGSATPHINAEMGDFADVVLMPGDPLRAKYIAETFLEDAREVNNVRGMLGFTGTYKGRKISVMGHG
MGIPSCSIYTKELITDFGVKKIIRVGSCGAVLPHVKLRDVVIGMGACTDSKVNRIRFKDHDFAAIADFDMVRNAVDAAKA
LGIDARVGNLFSADLFYSPDGEMFDVMEKYGILGVEMEAAGIYGVAAEFGAKALTICTVSDHIRTHEQTTAAERQTTFN
DMIKIALESVLLGDKE

Escherichia coli xanthosine phosphorylase (*Ec*XP, *Ec*XapA, WP_000283878.1) cloned via BamHI/HindIII sites from gene synthesis (GeneArt, FisherScientific)

ε_{280} = 13785 M^{-1} cm^{-1}, E1% = 4.4

MRGSHHHHHHGSYQAQFSHNPLYCVDIIKTYKPDFTPRVAFILGSGLGALADQIENAVAISYEKLPGFPVSTVHGHAG
ELVLGYLQGVPVACMKGRGHFYEGRGMTIMTDAIRTFKLLGCELLFCTNAAGSLRPEVGAGSLVALKDHINTMPGTP
MVGLNDERFGERFFSLANAYDAEYRALLQKVAKEEGFPLTEGVFVSYPGPNFETAAEIRMMQIIGGDVVGMSVVPEVI
SARHCELKVVAVSAITNMAEGLSDVKLSHAQTLAAAELSKQNFINLICGFLRKIA

Geobacillus thermoglucosidasius pyrimidine nucleoside phosphorylase (*Gt*PyNP, gene Geoth_1355, 7M7K_B or WP_041270053.1), cloned from gDNA as described in Szeker *et al*.[2]

ε_{280} = 21890 M^{-1} cm^{-1}, E1% = 4.6

MRGSHHHHHHGSMVDLIAKKRDGYELSKEEIDFIIRGYTNGDIPDYQMSAFAMAVFFRGMTEEETAALTMAMVRSGD
VIDLSKIEGMKVDKHSTGGVGDTTTLVLGPLVASVGVPVAKMSGRGLGHTGGTIDKLESVPGFHVEIDNEQFIELVNKN
KIAIIGQTGNLTPADKKLYALRDVTATVDSIPLIASSIMSKKIAAGADAIVLDVKTGAGAFMKDFAGAKRLATAMVEIGKR
VGRKTMAVISDMSQPLGYAVGNALEVKEAIDTLKGKGPEDLQELCLTLGSYMVYLAEKASSLEEARALLEASIREGKAL
ETFKVFLSAQGGDASVVDDPTKLPQAKYRWELEAPEDGYVAEIVADEVGTAAMLLGAGRATKEATIDLSVGLVLHKKV
GDAVKKGESLVTIYSNTENIEEVKQKLAKSIRLSSIPVAKPTLIYETIS

Human purine nucleoside phosphorylase was acquired from Sigma Aldrich (product number 540221, supplied as liquid enzyme preparation in 50 mM Tris-HCl, pH 7.6, with an activity of ≥25 units/mg protein, where “one unit is defined as the amount of enzyme that will convert 1 µmol of MESG* and 1 µmol of P_i** into 1 µmol of 2-amino-6-mercapto-7-methylpurine and 1 µmol of ribose phosphate in 1 min at 25°C, pH 7.6.”). *MESG is 7-methyl-6-thioguanosine, and **P_i is inorganic phosphate.

Transformations

We used a modified PCTUT7 vector, where expression is under control of a *lac*-operon and which introduces an *N*-terminal His_6-tag. This plasmid carries an ampicillin resistance cassette. To generate competent *E. coli* cells for the transformation with the respective constructs (see above), 5 mL LB medium (10 g L^{-1} tryptone, 5 g L^{-1} yeast extract, 10 g L^{-1} NaCl) were inoculated with *E. coli* BL21g and incubated at 200 rpm at 37 °C for 1 h. The cells were then pelleted at 4 °C in a pre-cooled table top centrifuge (Eppendorf) and the pellet resuspended in ice-cold 50 mM $CaCl_2$ (two aliquots of 600 µL) and incubated on ice for 30 min. The centrifugation was repeated, both pellets resuspended and combined in a total of 600 µL 50 mM $CaCl_2$ and incubated on ice for 2 h. Then, 100 µL of these chemically competent *E. coli* BL21g were transformed with 100 ng plasmid. To this end, the cells were incubated with the plasmid for 30 min on ice, followed by a heat shock at 42 °C for 2 min, and another incubation on ice for 5 min. Then, 1 mL SOC medium (0.186 g L^{-1} KCl, 0.5 g L^{-1} NaCl, 5 g L^{-1} yeast extract, 20 g L^{-1} tryptone, 10 mM $MgCl_2$, 10 mM $MgSO_4$ and 20 mM glucose) were added and the mixture was incubated at 37 °C and 700 rpm for 1 h. The resulting cells were then pelleted in a precooled centrifuge (4 °C), the supernatant was decanted (and discarded) and the remaining ≈100−150 µL of medium were used to plate the mixture onto LB plates (LB medium with 15 g L^{-1} agar) containing 100 µg mL^{-1} ampicillin and 1% glucose (to repress leaky expression from the plasmid). The plates were then incubated at 37 °C for 15−17 h and subsequently stored at 4 °C.

Protein production and purification

All enzymes were heterologously produced in *E. coli* as His_6-tagged proteins through IPTG-induced overexpression, as previously described.[3] For protein production, we employed two different strategies:

i) For production in Enpresso B medium (Enpresso GmbH, Berlin, Germany), which simulates a fed-batch culture in shake flasks, cells were washed from an agar plate of the expression strain (*E. coli* BL21g harboring the respective plasmid) with 1 mL 0.9 % NaCl using a spatula, according to the manufacturer's standard protocol. Then, OD_{600} was measured and 50 mL Enpresso B medium supplemented with antibiotic (100 mg L^{-1} ampicillin) was inoculated to an initial OD_{600} of 0.15. The resulting culture was grown at 37 °C and 250 rpm for 17 h before expression was induced by addition of isopropyl β-D-1-thiogalactopyranoside (IPTG) to a final concentration of 0.1 mM (50 µL of a 0.1 M stock). The culture was incubated at 30 °C and 250 rpm for 24 h before the cells were harvested by centrifugation (8000 g, 10 min, 4 °C). The resulting cell pellet was either stored at −20 °C until use or immediately subjected to lysis and purification.

ii) Alternatively, for *Gt*PNP and *Gt*PyNP, we employed a production workflow in traditional TB medium. To this end, a 20 mL preculture of the expression strain (*E. coli* JM109 harboring the respective plasmid) was grown in LB medium supplemented with antibiotic (50 mg L^{-1} ampicillin) overnight at 37 °C. This preculture was used to inoculate 250 mL TB medium (12 g L^{-1} tryptone, 24 g L^{-1} yeast extract, 5 g L^{-1} glycerol, 2.31 g L^{-1} KH_2PO_4, 12.54 g L^{-1} K_2HPO_4) containing antibiotic to an initial OD_{600} of around 0.15. This culture was incubated at 37 °C and 200 rpm until an OD_{600} of >0.6 was reached, which typically happened after 2−3 h. At this point, protein production was induced by adding IPTG to a final concentration of 0.1 mM (25 µL of a 1 M stock in water). Expression was carried out overnight (ca. 20 h) at 37 °C and cells were harvested by centrifugation (4000 g, 20 min, 4 °C). The resulting cell pellet was either stored at −20 °C until use or immediately subjected to lysis and purification.

For lysis and purification, pelleted cells were resuspended in binding buffer (20 mM KH_2PO_4, 500 mM NaCl, 20 mM imidazole) to a concentration of around 0.5 g_{pellet} mL^{-1} and Pierce™ protease inhibitor mini tables (Thermo Fisher Scientific) were added (one per 10 mL of suspension volume). Cells were disrupted by sonication (6 min, 10 s pulse, 10 s breaks, 60% amplitude or 10 min, 30 s pulse, 30 s breaks, 30% amplitude – both protocols performed similarly

well). For some enzymes, the resulting lysate was heated for 20−30 min to precipitate *E. coli* proteins (60 °C for *Gt*PNP and *Gt*PyNP, 80 °C for *Tt*PNP). Cell debris and precipitated protein was then removed by centrifugation (11000 rpm, 45 min, 4 °C) and filtration (0.45 µm pore size). The cell free extract was applied to a Ni Sepharose Histrap^{TM} column (GE Healthcare) preequilibrated with binding buffer. Non-specifically bound proteins were removed by washing with approximately 10 column volumes (CV) of binding buffer and the target protein was eluted with elution buffer (20 mM KH_2PO_4, 500 mM NaCl, 500 mM imidazole) over ca. 3 CV. Fractions containing pure target protein (as assessed by SDS PAGE) were combined and, if necessary, concentrated by centrifugation (Vivaspin, Sartorius, Göttingen, Germany, molecular weight cut-off at 10 kDa). Afterwards, the protein was desalted into 20 mM MOPS buffer (pH 7, adjusted at 20 °C) or 20 mM taurine buffer (pH 9, adjusted at 20 °C) using a PD-10 desalting column (GE Healthcare). This desalting procedure was repeated once (for a total of two passes through a PD-10 column per protein preparation). Alternatively, the target protein obtained from the affinity column was directly dialyzed against 2 mM phosphate buffer (pH ≈9). The resulting protein preparations were stored at 4 °C. Alternatively, glycerol was added to a final concentration of 50% (v/v) to store the resulting protein stocks at −20 °C. Typical stock concentrations of pure protein ranged from 1−10 g L^{-1} (calculated with 1 AU at 280 nm being equal to a protein concentration of 1 g L^{-1}).

Discovery of N7X (Figs. 1c and 1d)

We first discovered **N7X** via its unusual UV spectroscopic properties.

In a reaction cascade featuring a purine nucleoside phosphorylase (purine NP, converting guanosine to guanine) and a deaminase (converting guanine to xanthine), we recorded UV spectra which did not match the expected products.

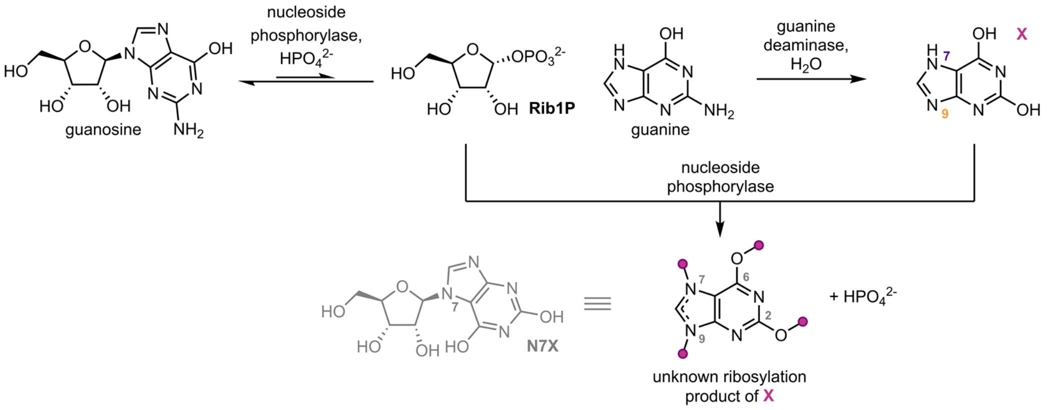

Supplementary Figure 1. Discovery of **N7X** as an unexpected product of a biocatalytic cascade converting of guanosine to **X**.

For instance, we performed reactions with 200 µM guanosine, 400 µM phosphate, 100 µg mL^{-1} (ca. 3.8 µM) *Gt*PNP and 1 µg mL^{-1} deaminase in 50 mM glycine buffer pH 9 in a total volume of 500 µL at 40 °C for 2 h. Then, 100 µL of reaction mixture were quenched in 100 µL 200 mM NaOH directly in wells of a UV-transparent 96-well plate. Subsequently, we recorded UV absorption spectra from 250 to 350 nm in steps of 1 nm with a platereader. Reference spectra for **X** and **N9X** were obtained from solutions of either compound in 50 mM glycine buffer and analogous dilution in aq. NaOH. This gave spectra such as those shown in Fig. 1c.

To check for the potential formation of glycosides of **X**, an analogous reaction mixture to the one described above was quenched 1:1 with MeOH, centrifuged to remove any precipitate (13,000 rpm, 10 min), and subjected to HRMS analysis (negative mode, as all relevant components in this mixture are anions at pH 9). This yielded clear evidence for the formation of a glycoside (Fig. 1d) with ions corresponding to xanthosine (**N9X**) or any of its isomers.

HPLC analyses (Fig. 1e)

To further exclude the formation of **N9X** by *Gt*PNP at pH 9, we analyzed the reaction products by HPLC. To this end, we performed a reaction containing 500 µM **X**, 500 µM **Rib1P**, and 1 g L^{-1} *Gt*PNP in 50 mM glycine-KOH buffer pH 9. This reaction was started by the addition of the enzyme and incubated at 50 °C for 24 h. Afterwards, a 50 µL sample of the reaction mixture was quenched by addition to 50 µL ice cold methanol. The resulting sample was centrifuged (13,000 rpm, 10 min) and analyzed by HPLC, using an Agilent 2200 system with DAD detector at 260 nm. The eluents were a) 50 mM ammonium-acetate buffer pH 5 (from a 1 M stock adjusted to the correct pH with 100 % acetic acid) and b) MeCN. The column was a 250x4.6 5µm Kinetex Evo C18 Core Shell (Phenomenex), used at a flow rate of 1 mL min^{-1} and heated to 25 °C. The elution method consisted of a linear gradient from 3−40% MeCN over 10 min, followed by 4 min of re-equilibration at 3% MeCN. This experiment provided further evidence that the reaction product of the *Gt*PNP-catalyzed glycosylation of **X** at pH 9 was not **N9X**. Indeed, under these HPLC conditions, the typical

retention times were 3.4−3.5 min (**X**), 4.3−4.4 min (**N7X**), and 4.5−4.6 min (**N9X**), as confirmed with authentic standards of **X** and **N9X**, and purified **N7X**.

Activity screening of *Gt*PNP for production of N7X

To evaluate the relative activity of *Gt*PNP for the production of **N7X** at different pH values, we performed kinetic experiments monitored by HPLC. To this end, we performed reactions containing 800 µM **X**, 800 µM **Rib1P**, and *Gt*PNP (1.15−3.60 µg mL^{-1}) in phosphate-hydrazine-borate buffer[4] (50 mM of each component) at pH 5, 6, 7, 8, and 9 at 50 °C in a total volume of 450 µL. These reactions were started by addition of the appropriately diluted enzyme (16 µL) to the rest of the reaction mixture (434 µL). Each enzyme concentration was chosen so that the conversion would be below 10 % after ca. 30 min so that we could take five samples over that reaction time. These samples (50 µL each) were quenched and analyzed by HPLC as described above. Due to the lack of an authentic standard for **N7X**, conversion was calculated based on peak areas which were converted to concentrations using response factors measured with authentic standards for **X** and **N9X**. The reaction courses were fitted with the first-order rate equation (1).

$$TN = TN_{eq} - TN_{eq}\exp\left(-k''_{obs}\, t\right) \qquad (1)$$

where TN is the turnover number (molecules of product generated per active site, dimensionless), TN_{eq} is the turnover number in the apparent equilibrium (also dimensionless), k''_{obs} is the observed first-order rate constant expressing the rate to reach equilibrium (min^{-1}) and t is the reaction time (min). Multiplication of k''_{obs} and TN_{eq} then yields the rate constant k'_{obs} expressing the number of turnovers per enzyme per minute. These fits yielded k'_{obs} = 66.5 $\pm$ 4.3 min^{-1} and TN_{eq} = 1742 (R^2 = 0.995) for pH 5, k'_{obs} = 58.5 $\pm$ 2.8 min^{-1} and TN_{eq} = 1428 (R^2 = 0.997) for pH 6, k'_{obs} = 90.1 $\pm$ 3.5 min^{-1} and TN_{eq} = 1305 (R^2 = 0.994) for pH 7, k'_{obs} = 29.9 $\pm$ 1.4 min^{-1} and TN_{eq} = 483 (R^2 = 0.991) for pH 8, and k'_{obs} = 6.8 $\pm$ 0.6 min^{-1} and TN_{eq} = 105 (R^2 = 0.992) for pH 9. These experiments clearly indicated that *Gt*PNP is more active in the synthesis of **N7X** at lower pH values, although this does erode the selectivity of the transformation as lower pH values also yield **N9X**. However, the strong pH-dependence of TN_{eq} indicates that either the equilibrium constant is pH-dependent or there are severe inhibition effects at play – both are likely scenarios. We also cannot exclude that **N7X** may have formed borate esters under these conditions.[5]

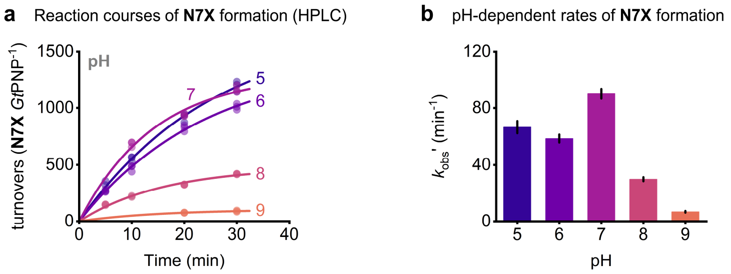

Supplementary Figure 2. pH-dependent kinetics of **N7X** formation by *Gt*PNP as monitored by HPLC. The errors in panel **b** display the fitting errors in **a**.

Computational prediction of UV absorption spectra (Fig. 1f)

To narrow down possible glycosylation isomers of **X**, we first predicted UV spectra for all likely candidates by density functional theory (DFT). To this end, DFT calculations for all structures (Chart S1) were performed using the Amsterdam Modeling Suite (AMS) 2020.203.[6] For initial geometry optimizations the PBE[7] XC functional with a TZP slater-type orbital basis set[8] was applied and the COSMO[9] model was used for solvation in water. The subsequent time-dependent DFT (TDDFT) single-point calculations were achieved using the B3LYP[10–12] hybrid functional with a QZ4P basis set.[8] Solvation in water was again performed by COSMO and excitation energies were calculated according to the Davidson algorithm.[13] The generation of UV/Vis spectra was achieved in Python. For each spectrum, the contribution of the different calculated structures to the spectrum was determined based on the corresponding electronic energy applying the Boltzmann equation. The final spectra were subsequently convoluted applying peak broadening with Gaussians. The most redshifted peak of each spectrum was then normalized to its maximum and plotted as shown in Fig. 1f.

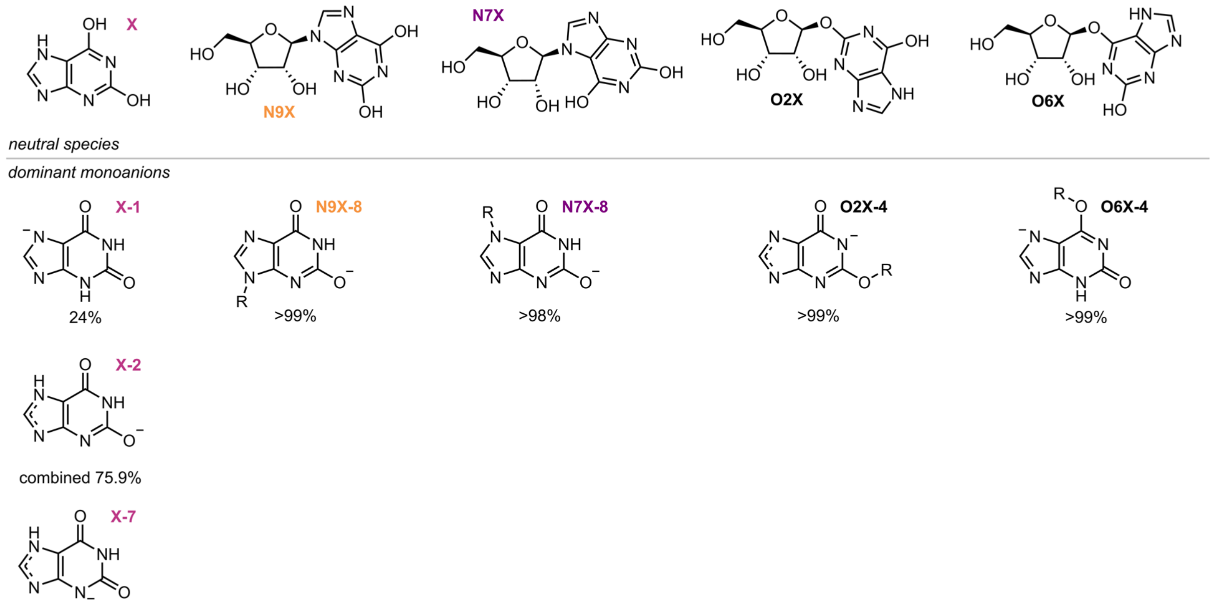

Supplementary Figure 3. Dominant anionic tautomers of **X** and its glycosides. The numbers correspond to the ones used in the externally hosted supplementary information and during calculations. Please see the zenodo entry for the full list of considered tautomers, code and calculation results.[14]

Purification of N7X and NMR analyses (Fig. 1g)

N7X was produced by *Gt*PNP-catalyzed glycosylation of **X** and purified by semi-preparative HPLC. To this end, 18.3 mg **X**, 119.2 mg **Rib1P**, and *G*tPNP (0.1 g L^{-1}) were combined in 12 mL 50 mM HEPES buffer pH 7 (although pH 7 does decrease the stability of **N7X** in the presence of *G*tPNP, see below, this enzyme is much more active in this transformation than at pH 9, see above). This equals final concentrations of 10.5 mM **X** and 21 mM **Rib1P** in the reaction. The reaction mixture was incubated at 60 °C for 5 h and then frozen to stop the reaction. Next, the mixture was thawed and the denatured enzyme and precipitated (crystalline) **X** were removed by centrifugation at 4 °C. **N7X** was then purified by semi-preparative HPLC using a 250x10 mm MultoKrom100-10 C18 column (Chromatographie Service GmbH, Langerwehe, Germany). The purification method used water (ice-cold) and MeOH (room temperature) as eluents, a flow rate of 4.7 mL min^{-1} and an elution program starting with isocratic elution with 3% MeOH for 5 min, followed by a linear gradient to 10% MeOH over 2.5 min (7.5 min run time) and a drop back down to 3% MeOH over 0.5 min (8 min run time), which was held for 6 min (14 min run time) to re-equilibrate the column. Per run, 2 mL of sample were injected, for a total of 6 runs. **N7X** typically eluted between 3 and 5 min of run time, followed by **X** between 5.5 and 7.5 min. **N9X** was not detected in this experiment. This yielded a total of 12 mg partially purified **N7X**, which contained **Rib1P** and HEPES as major impurities (detected by NMR) due to its early elution from HPLC. Of this sample, 8 mg were dissolved in D_2O (containing 0.05 wt% TSP as internal standard) for NMR analysis. Following the tentative identification of the nucleoside product as **N7X** (see below for structure elucidation), this NMR sample was diluted with water, freeze-dried and triturated with DMSO-d_6, which dissolved **N7X**, some of the HEPES, while leaving most of the **Rib1P** untouched. NMR analysis of the resulting sample unambiguously identified the product as **N7X** due to matching data with Bridson's report (Supplementary Table 3).[15] All NMR data collected in this study are freely available from the externally hosted supplementary information.[14] For clarity, parts of the data are highlighted below.

Structure elucidation of N7X

We elucidated the structure of **N7X** by combined 1D and 2D NMR from a partially purified sample in D_2O. The major impurities in our sample were **Rib1P** and HEPES. ^1H and ^{13}C NMR confirmed the conclusion from HRMS by revealing the presence of a ribosyl unit (δ_H = 6.15, 4.59, 4.32, 4.22 and 3.95/3.84 ppm) and an **X** unit (quaternary carbons with δ_C = 159.4, 155.4, 153.4, 144.9 and 109.8 ppm and a sole proton at 8.29 ppm). The relatively high shift of the anomeric proton of the ribosyl unit (6.15 ppm) and its multiplicity (d with J = 4.2 Hz) indicated glycosylation of a heteroatom on an aromatic system. Next, aided by reference data for **X** in C_6D_6 reported by Nicolau and Hildenbrand,[16] HSQC and HMBC allowed full assignment of all carbons and protons in the molecule (see Supplementary Table 1 below). HMBC correlations between H-1′ and C-8 as well as to C-5 placed the ribosylation site at the imidazole unit of **X**. Since the product cannot be **N9X** (as clearly shown by UV and retention time on HPLC), that leaves only *N*7. The lack of a HMBC correlation to C-4 is also in line with ribosylation at *N*7. Since glycosylation of *N*7 places H-8 in the spatial vicinity of the top face of the ribosyl unit where it is also fixed by a hydrogen bond to *O*6 (also see Fig. 1g), we expected to see (at least weak) NOESY correlations between H-8 and H-1′, H-2′, and H-3′, which, indeed, we did. These data unambiguously identify the glycosylation product as **N7X**. The tabulated NMR data are available below and the full set of raw NMR data (^1H, ^{13}C, COSY, NOESY, HMBC, and HSQC in D_2O and ^1H, COSY, HSQC, and HMBC in DMSO-d_6) are available for download from zenodo.org.[14]

Following the suggestions by the two very constructive reviewers, we subjected our sample of **N7X** to additional purification. To this end, the partially purified sample of **N7X** in DMSO-d_6 was applied to C18 silica, which was washed with water before **N7X** was eluted with a gradient of 0% to 5% MeCN over 6 CV. Fractions containing **N7X** were identified by UV (254 nm), combined and dried to give ca. 0.5 mg **N7X**. The ^1H NMR data of this material agree with

those of the partially purified material and are provided below (Supplementary Fig. 7). These data were also used to supplement some of the poorly resolved signals in Supplementary Tabs. 2 and 3.

Supplementary Table 1. ^{1}H (700 MHz) and ^{13}C (176 MHz) NMR data of **N7X** in D_2O

Position	δ_H (mult., *J* in Hz)	COSY correlations	δ_C (type)	^{1}H,^{13}C HMBC correlations
2			155.9 (Cq)	
4			159.4 (Cq)	
5			109.8 (Cq)	H-8, H-1′ (weak)
6			153.4 (Cq)	H-8
8	8.29 (s)		144.9 (CH)	H-8, H-1′
1′	6.15 (d, 4.2)	2′	93.0 (CH)	H-1′, H-2′ (weak), H-3′
2′	4.59 (dd, 5.2, 4.3)	1′, 3′	77.4 (CH)	H-1′
3′	4.32 (dd, 5.4, 4.5)	2′, 4′	72.0 (CH)	H-3′, H-4′, H-5′
4′	4.22 (m)	3′, 5′, 5′	87.4 (CH)	H-2′ (weak), H-5′ (weak)
5′	3.95 (dd, 12.8, 3.1)<br>3.84 (dd, 12.8, 4.7)	4′	63.7 (CH_2)	H-3′

Supplementary Table 2. ^{1}H (700 MHz) and ^{13}C (176 MHz) NMR data of **N7X** in DMSO-d_6 (referenced to TMS)

Position	δ_H (mult., *J* in Hz) (type)	COSY correlations	δ_C*	$^1H,^{13}C$ HMBC correlations
1	10.96 (s) (N-H)**			
2				
3	11.66 (br s) (N-H)**			
4				
5			108.0	H-8, H-1′
6			151.8	H-8
8	8.36 (s)		142.9	H-8, H-1′
1′	6.02 (d, 5.1)	2′	90.6	H-1′, H-1′-OH
2′	4.34 (q, 5.1)<br>5.46 (d, 5.9) (O-H)	2′-OH, 3′	76.3	
3′	4.09 (q, 4.9)<br>5.16 (d, 5.0) (O-H)	3′-OH, 4′	71.1	
4′	3.91 (q, 4.0)	5′	86.8	
5′	3.68 (ddd, 12.0, 5.4, 4.1)<br>3.55 (ddd, 12.0, 5.6, 4.1)<br>5.04 (t, 5.6 Hz) (O-H)	5′-OH<br>5′-OH	62.3	

*inferred from HSQC/HMBC correlations due to the low solubility of the compound. Some signals are missing.
**reported in analogy to Bridson *et al.*[15] who assigned these protons as N-H. However, due to tautomerism, these may also share some O-H character. The excessive broadening of these protons in wet DMSO-d_6 provides some indication of relevant tautomerism, in particular for H-3.

Supplementary Table 3. Comparison of ^{1}H NMR data with Bridson's[15] synthetic sample of **N7X** (both in DMSO-d_6 rel. to TMS)

Position	δ_H<br>Synthetic sample<br>(300 MHz) (mult.)	δ_H<br>Biocatalytic sample<br>(700 MHz) (mult., *J*)	Δppm if referenced to TMS<br>(if referenced to res. solvent)
1 (N-H)	10.96 (s, 1H)	10.96 (s, 1H)	0.00 (−0.02)
3 (N-H)	11.66 (br s, 1H)	11.66 (br s, 1H)	0.02 (−0.00)
8	8.35 (s, 1H)	8.36 (s, 1H)	0.01 (−0.01)
1′	6.01 (d, 1H)	6.02 (d, *J* = 5.1 Hz, 1H)	0.01 (−0.01)
2′	4.32 (m, 1H)	4.34 (q, *J* = 5.1 Hz, 1H)	0.02 (0.00)
2′-OH (O-H)	5.44 (d, 1H)	5.46 (d, 5.7 Hz, 1H)	0.02 (0.00)
3′	4.08 (m, 1H)	4.09 (q, *J* = 4.9 Hz, 1H)	0.01 (−0.01)
3′-OH (O-H)	5.14 (d, 1H)	5.16 (d, 4.6 Hz, 1H)	0.02 (0.00)
4′	3.90 (m, 1H)	3.91 (q, *J* = 4.0 Hz, 1H)	0.01 (−0.01)
5′	3.60 (m, 2H)	3.68 (ddd, *J* = 12.0, 5.4, 4.1 Hz, 1H)<br>3.55 (ddd, *J* = 12.0, 5.6, 4.1 Hz, 1H)	0.02 (0.00)
5′-OH (O-H)	5.02 (t, 1H)	5.04 (t, 5.0, 1H)	0.02 (0.00)

Supplementary Table 4. Comparison of key ^{1}H (700 MHz) and ^{13}C (176 MHz) NMR data of **X**, **N7X** and **N9X** in D_2O*,**

Pos.	**X^-***Na^+*		**N7X**		**N9X** / ***N9X**$^-$**Na**$^+$*	
	δ_H (mult., *J* in Hz)	δ_C	δ_H (mult., *J* in Hz)	δ_C	δ_H (mult., *J* in Hz)	δ_C
2				155.9		
4				159.4		
5		*116.0*		109.8		118.7<br>*118.2*
6		*157.4*		153.4		149.9<br>*155.7*
8	*7.74 (s)*	*141.8*	8.29 (s)	144.9	7.98 (s)<br>*7.87 (s)*	140.0<br>*140.1*
1′			6.15 (d, 4.2)	93.0	5.87 (d, 6.5)<br>*5.86 (d, 6.3)*	91.4<br>*90.8*
2′			4.59 (dd, 5.2, 4.3)	77.4	4.66 (dd, 6.5, 5.2)<br>*4.76 (m)*	76.4<br>*76.1*
3′			4.32 (dd, 5.4, 4.5)	72.0	4.39 (dd, 5.3, 2.9)<br>*4.41 (m)*	73.6<br>*73.7*
4′			4.22 (m)	87.4	4.28 (q, 2.9)<br>*4.26 (m)*	88.7<br>*88.7*
5′			3.95 (dd, 12.8, 3.1)<br>3.84 (dd, 12.8, 4.7)	63.7	3.91 (dd, 12.8, 2.7)<br>*3.91 (m)*<br>3.85 (dd, 12.8, 2.9)<br>*3.81 (m)*	64.2<br>*64.4*

*The sodium salts were recorded by adding a drop of 10 M NaOH to the NMR sample in D_2O. Neutral **X** was essentially insoluble.

^{1}H NMR, ^{1}H,^{13}C HSQC and ^{1}H,^{13}C HMBC spectra for **X^-, **N9X** and **$N9X^-$** are available from the externally hosted supplementary information.[14]

Reference UV spectra of X and N7X at pH 10 (Fig. 1h)

At pH 10, **X** and **N7X** almost completely exist as their anions (see Fig. 1i). We therefore obtained reference spectra of both compounds at pH 10 to enable continuous reaction monitoring (see below). To this end, we prepared a 100 µM solution of **X** in 50 mM glycine/proline buffer pH 10 and measured its UV absorption spectrum in a platereader using UV-transparent 96-well plates. The Lambert-Beer law directly provided the extinction coefficients of **X** at all wavelengths. Since accurate preparation of exact concentrations of **N7X** proved difficult, we used isosbestic normalization based on the known isometric point of glycosylation to correct for concentration errors. Thus, we prepared a ca. 100 µM solution of **N7X** (purified as described above) and obtained its UV absorption spectrum in analogy to **X**. Next, we normalized the spectra of **X** and **N7X** to the isosbestic point of glycosylation (288 nm) and used the scaling factor between the extinction coefficient of **X** and the normalized spectrum of **X** to calculate the extinction coefficients of **N7X** based on the normalized spectrum of **X**. The result is shown Fig. 1h and all experimental and calculated data are available from the externally hosted supplementary information.[14]

Absorption spectra of X, N9X and N7X as functions of pH (Fig. 1i)

To determine the pK_a values of **X**, **N9X** and **N7X**, we obtained UV absorption spectra from pH 2−12 and applied isosbestic normalization for fitting. To this end, we prepared ≈100 µM solutions of each compound in a universal buffer mix containing citrate (pK_a = 3.1, 4.8, and 6.4), HEPES (pK_a = 3.0 and 7.5), asparagine (pK_a = 2.1 and 8.8) and proline (pK_a = 2.0 and 10.6) (all adjusted to the respective pH with HCl and NaOH, all with 5 mM final concentration of each buffer component) and obtained UV absorption spectra of these solutions from 250 to 350 nm in steps of 1 nm in a platereader using UV-transparent 96-well plates. This revealed isosbestic points of deprotonation of 274 nm (**X**), 259/268 nm (**N9X**), and 279 nm (**N7X**). All spectra were then normalized to the absorbance at the isosbestic point (Supplementary Fig. 4) and the distribution of the neutral and deprotonated species was calculated as a function of pH through equation (2).[3]

$$\alpha = \frac{A_{\lambda,n,i} - A_{\lambda,n,min}}{A_{\lambda,n,max} - A_{\lambda,n,min}} \quad (2)$$

where α is the molar fraction of the deprotonated species (dimensionless), $A_{\lambda,n,i}$ is the normalized absorbance obtained for the sample i at the arbitrary reference wavelength λ, $A_{\lambda,n,min}$ is the minimal normalized absorbance obtained at λ ($\alpha \approx 0$), and $A_{\lambda,n,max}$ is the maximal normalized absorbance obtained at λ ($\alpha \approx 1$). Fitting of equation (3) then provided the pK_a values of **X**, **N9X**, and **N7X**.[17]

$$\alpha = \frac{10^{pH-pK_a}}{1 + 10^{pH-pK_a}} \quad (3)$$

where pH is the pH value at which α was obtained and pK_a is the acid dissociation constant of the compound. This analysis yielded pK_a = 7.28 ± 0.06 for **X** (R^2 = 0.994), pK_a = 5.31 ± 0.01 for **N9X** (R^2 = 0.999), and pK_a = 8.47 ± 0.07 for **N7X** (R^2 = 0.989), using arbitrarily chosen wavelengths near the maximum of the anion of each species (286 nm for **X**, 281 nm for **N9X**, and 294 nm for **N7X**) as a reference wavelengths λ. These fits are shown in Figure 1i in the main text.

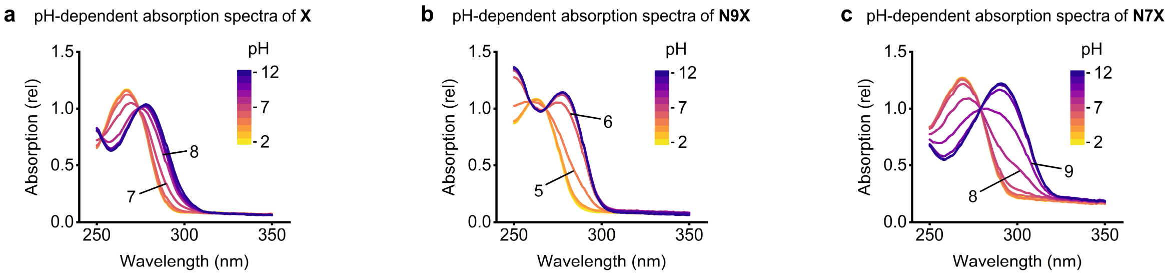

Supplementary Figure 4. Normalized UV absorption spectra of **X** (**a**), **N9X** (**b**), and **N7X** (**c**) at different pH values.

Equilibrium constant of phosphorolysis of N7X (Figs. 1j and 1k)

To determine the equilibrium constant of phosphorolysis of **N7X**, we performed glycosylation experiments monitored by UV. To this end, we continuously monitored the glycosylation of **X** catalyzed by *Gt*PNP at pH 10, where **X** and **N7X** almost exclusively exist as monoanions and *Gt*PNP selectively yields **N7X** (and not **N9X**). Specifically, we performed glycosylation reactions with **X** (100 or 150 µM), **Rib1P** (0, 25, 50, 100, 200 or 500 µM, equivalent to 0, 0.25, 0.5, 1, 2 and 5 or 0.16, 0.33, 0.66, 1.33 and 3.33 equivalents) and 400 µg mL^{-1} *Gt*PNP (equivalent to ca. 15.4 µM) in 50 mM glycine/proline buffer (containing 50 mM of both buffer components) pH 10 in wells of a UV-transparent 96-well plate in a total volume of 200 µL at room temperature (ca. 21 °C). These reactions were started by addition of 150 µL reaction mixture (containing all reagents except **Rib1P**) to 50 µL appropriately diluted solution of **Rib1P**. These reactions were monitored at 288 nm (the isosbestic point of phosphorolysis of this transformation at pH 10) and 300 nm (near the maximum positive difference of the extinction coefficients, $\Delta\varepsilon_{300}$ = 3.6 mM^{-1} cm^{-1}) for 4 h. The resulting absorption data were converted to conversions via equation (4)[3] (also see our tutorial paper[18] for details).

$$c = \frac{\mathrm{A}_{300/288} - \mathrm{A}_{300/288,\mathbf{X}}}{\mathrm{A}_{300/288,\mathbf{N7X}} - \mathrm{A}_{300/288,\mathbf{X}}} \qquad (4)$$

where c is the conversion of **X** to **N7X**, $\mathrm{A}_{300/288}$ is the ratio between the observed absorbances at 300 nm and 288 nm, $\mathrm{A}_{300/288,\mathbf{X}}$ is the 300 nm/288 nm ratio of the nucleobase **X**, and $\mathrm{A}_{300/288,\mathbf{N7X}}$ is the 300 nm/288 nm ratio of the nucleoside **N7X**. All these variables are dimensionless and all absorbances are background-corrected. We determined the reference values $\mathrm{A}_{300/288,\mathbf{X}}$ and $\mathrm{A}_{300/288,\mathbf{N7X}}$ from reference spectra of both compounds obtained at pH 10 in 50 mM glycine/proline buffer ($\mathrm{A}_{300/288,\mathbf{X}}$ = 0.22 and $\mathrm{A}_{300/288,\mathbf{N7X}}$ = 0.85). Treatment of the raw data with equation (4) yielded reaction courses (e.g. those shown in Fig. 1j, the rest is available from the externally hosted supplementary information).[14] The equilibrium conversions of these reactions were then plotted as a function of the excess of **Rib1P** (in equivalents) and fitted with equation (5) to obtain the equilibrium constant of glycosylation as defined by equation (6), which was converted to the equilibrium constant of phosphorolysis through equation (7). Please see our previous publication on nucleoside diversification[19] for the derivation of this equation in the phosphorolytic direction.

$$c_{eq} = \frac{-K_{gly} - K_{gly}x + \sqrt{\left(K_{gly} + K_{gly}x\right)^2 + 4K_{gly}x(1 - K_{gly})}}{2 - 2K_{gly}} \qquad (5)$$

$$K_{gly} = \frac{[\mathbf{N7X}][\mathrm{P}]}{[\mathbf{X}][\mathbf{Rib1P}]} \qquad (6)$$

$$K_{phos} = \frac{1}{K_{gly}} \qquad (7)$$

where c_{eq} is the extent of conversion in the equilibrium (in fractions, not in percent), K_{gly} is the equilibrium constant of phosphorolysis as defined by (6) (dimensionless), x is the excess of **Rib1P** over **X** (in fractions), $[\mathbf{N7X}]$ is the concentration of **N7X**, $[\mathrm{P}]$ is the concentration of phosphate, $[\mathbf{X}]$ the concentration of **X** and $[\mathbf{Rib1P}]$ the concentration of ribose 1-phosphate (all arbitrary molar concentrations), and K_{phos} is the equilibrium constant of phosphorolysis

(dimensionless). Fitting of the experimental data to equation (5), as shown in Fig. 1k (while excluding the datapoint for 5 equivalents), yielded K_{gly} = 1.85 ± 0.23 (R^2 = 0.983). Therefore, K_{phos} = 0.54 ≈ 0.5.

Michaelis-Menten kinetics with *Gt*PNP (Fig. 1l)

To evaluate the affinity of *Gt*PNP toward **X** under alkaline conditions (which essentially exclusively give **N7X**), we obtained Michaelis-Menten kinetics of this transformation at pH 10. To this end, we performed glycosylation reactions with **X** (20−500 µM), 200 µM **Rib1P** and 100 µg mL^{-1} *Gt*PNP (equivalent to ca. 3.8 µM) in 50 mM glycine/proline buffer (containing 50 mM of both buffer components) pH 10 in wells of a UV-transparent 96-well plate in a total volume of 200 µL at room temperature (ca. 21 °C). These reactions were started by addition of 100 µL reaction mixture (containing all reagents except **X**) to 100 µL appropriately diluted solution of **X**. All reactions were run duplicate and repeated with duplicates run with 50 µg mL^{-1} *Gt*PNP (1.9 µM), for a total of four replicates per concentration of **X**. Reaction progress was monitored at 300 nm ($\Delta\varepsilon_{300}$ = 3.6 mM^{-1} cm^{-1}) for 20 min. The absorption change over time was approximated by linear fitting (no forced intercept) and converted into observed rate constants with equation (8).

$$k_{obs} = \frac{m}{\Delta\varepsilon \, \mathrm{d} \, [E]} \tag{8}$$

where k_{obs} is the observed rate constant (min^{-1}), m is the linear slope of the absorption observed at a given wavelength (min^{-1}; in this case at 300 nm), Δε is the difference in extinction coefficient between **N7X** and **X** (3.6 mM^{-1} cm^{-1} at 300 nm and pH 10), d is the path length (generally 1 cm or corrected to 1 cm) and $[E]$ is the enzyme concentration (µM; our 10 g L^{-1} working stock of *Gt*PNP had a concentration of 384 µM). Given that stoichiometry holds true, this analysis treats substrate consumption and product formation rate equally. The observed rate constants as a function of the input substrate concentration were fitted to the Michaelis-Menten equation (9).

$$k_{obs,max} = \frac{k_{obs} \, [S]}{K_M + [S]} \tag{9}$$

where $k_{obs,max}$ is the maximum observed rate constant (equivalent to k_{cat}; min^{-1}), k_{obs} is the rate constant (min^{-1}) observed at the substrate concentration $[S]$ (mM, in this case $[\mathbf{X}]$) and K_M is the Michaelis-Menten constant (mM). Fitting of the resulting data to equation (9) yielded $k_{obs,max}$ = 6.09 ± 2.01 min^{-1} and K_M = 2.85 ± 1.08 mM (R^2 = 0.991).

Since the kinetic data only describe a very slight curvature, we additionally performed an analysis via the Akaike information criterion (AIC) to confirm our choice of a Michaelis-Menten fit over a simple linear approximation. To this end, we fitting our kinetic data again with the linear equation (10) instead of (9).

$$k_{obs} = a \, [\mathbf{X}] \tag{10}$$

where definitions from above apply and a is the slope (min^{-1} mM^{-1}). This fit yielded a = 1.85 ± 0.03 min^{-1} μM^{-1} (R^2 = 0.994). We then obtained AICs through Originlab's *Compare Models* function. This gave AICs of −135.4 for equation (9) and −130.8 for equation (10), clearly supporting the Michaelis-Menten model as the better fit of the kinetic data. All raw UV traces for this experiment are available from the externally hosted supplementary information.[14]

Synthesis of N7X/N9X by various nucleoside phosphorylases (Fig. 1m)

To examine the ability of various NPs to synthesize **N7X**, we screened a small but diverse selection of NPs. To this end, we performed glycosylation reactions with 500 µM **X**, 500 µM **Rib1P**, and 1 g L^{-1} NP in 50 mM glycine-KOH buffer at pH 9 or 50 mM HEPES buffer at pH 7. This reaction was started by the addition of the enzyme and incubated at 50 °C for 1 or 24 h. Afterwards, a 50 µL sample of the reaction mixture was quenched by addition to 50 µL ice cold methanol and analyzed by HPLC as described above. For this experiment, we used bacterial (purine and pyrimidine NP from *Geobacillus thermoglucosidasius*, purine NP from *Escherichia coli*, and purine NP1 and NP2 from *Thermus thermophilus*) and mammalian NPs (human purine NP). This yielded the conversions as listed in Supplementary Table 5.

Supplementary Table 5. Time- and pH-dependent formation of **N7X** and **N9X** by NPs

Enzyme*	Conversion to **N7X** (1 / 24 h) (%)		Conversion to **N9X** (1 / 24 h) (%)	
	pH 7	pH 9	pH 7	pH 9
*Gt*PNP	47 / <1	35 / 17	1 / 5	<1 / 1
*Gt*PyNP	24 / 43	13 / 40	5 / 17	3 / 10
*Tt*PNP	16 / 10	6 / 3	49 / 30	62 / 61
*Tt*PNP II	26 / 18	<1 / <1	6 / 22	<1 / <1
*Ec*PNP	23 / 5	<1 / 5	<1 / 2	<1 / <1
*Ec*XP	4 / 35	<1 / <1	62 / 56	70 / 74
Human PNP	2 / 9	<1 / <1	34 / 23	37 / 37
Control**	<1 / <1	<1 / <1	<1 / <1	<1 / <1

*for the PNPs: preferentially makes **N7X**, preferentially makes **N9X**.

**Performed with cell lysate of *E. coli* not expressing any His-tagged enzyme, which was subjected to the same purification procedure as the heterologously expressed enzymes

The preference of these enzymes for either **N7X** or **N9X** seems to be largely determined by the primary nucleobase-coordinating residue, which is either an asparagine (*Tt*PNP I, *Ec*PNP and human PNP – these enzymes preferentially make **N9X**) or an aspartate (*Gt*PNP, *Tt*PNP II and *Ec*PNP – these enzymes preferentially make **N7X**). Sequence alignment with Clustal Omega (see next page) illustrates this key difference. We hypothesize that different H-bonding behavior of **X** in either environment (with Asn or Asp) determines productive positioning of the nucleoside for formation of the nucleoside (**N7X** or **N9X**), as these enzymes primarily bind the nucleobase by C-H-π interactions with a key phenylalanine and H-bonding to Asn/Asp. Known crystal structures (e.g. 1rct or 1pf7) nicely illustrate this binding mechanism. Stachelska-Wierzchowska *et al.*[20] have proposed a similar mechanism underlying the switch in selectivity of *E. coli* PNP D204N and calf PNP N243D, which presumably lead to the formation of ribosylation isomers of 8-azaguanosine.

```
TtPNP_I         -------MM--------------------------------RGSHHHHHHGSSP--IHVR     19
GtPNP           -------MM--------------------------------RGSHHHHHHGSLS--IHIE     19
EcPNP           -------MM--------------------------------RGSHHHHHHGSAT--PHIN     19
EcXP            MRGSHHHHHHGSYQAQ-FSHNPLYCV------DIIKTYKPDFTPRVAFILGSGLGALADQ     53
TtPNP_II        -------MMRGSHHHHHHGSPDMELYDKIQEAVAYVRSKTDFVPEVGLVLGSGLGPLADE     53
human_PNP       --------------------ENGYTYEDYKNTAEWLLSHTKHRPQVAIICGSGLGGLTDK     40
                                                            .     **       .

TtPNP_I         AHPGDVAERVLLPG-----DPGRAEWIAKTFLQNPRRYNDHRGLWGYTGLYKGVPVSVQT     74
GtPNP           AKQQEIAEKILLPG-----DPLRAQYIAETFLEGATCYNRVRGMLGFTGTYKGHRISVQG     74
EcPNP           AEMGDFADVVLMPG-----DPLRAKYIAETFLEDAREVNNVRGMLGFTGTYKGRKISVMG     74
EcXP            IENAVAISYEKLPGFPVSTVHGHAGELVLGYLQGVPVA-----------CMKGRGHFYEG     102
TtPNP_II        VEKVAEIPYGEIPHFPVSTAPGHAGRLVLGRLEGKPVL-----------VYKGRVHYYEG     102
human_PNP       LTQAQIFDYSEIPNFPRSTVPGHAGRLVFGFLNGRACV-----------MMQGRFHMYEG     89
                           :*         :*  :.   *:.                 :*

TtPNP_I         TGMGTPSAAIVVEELV-RLGARVLVRVGTAGAASSDLAPGELIV---------AQGAVPL     124
GtPNP           TGMGVPSISIYVNELIQSYHVQTLIRVGTCGAIQKDVNVRDVIL---------AMSASTD     125
EcPNP           HGMGIPSCSIYTKELITDFGVKKIIRVGSCGAVLPHVKLRDVVI---------GMGACTD     125
EcXP            RGMTIMTDAIRT---FKLLGCELLFCTNAAGSLRPEVGAGSLVALKDHIN---TMPGTPM     156
TtPNP_II        YSAEEVVFPVRV---GFFLGARTFLLTSAAGGLNPRFRAGGIMLHLDYI---NFAGANPL     156
human_PNP       YPLWKVTFPVRV---FHLLGVDTLVVTNAAGGLNPKFEVGDIMLIRDHINLPGFSGQNPL     146
                         : .           :.  ..:.*.    .    ::

TtPNP_I         DGTTRQYLEGRPYAPVPDP--EVFRALWRR--AEALGYPHRVGLVASEDAFYATTPEEAR     180
GtPNP           SNMNRLTFRGRDYAPTANF--ALLRTAYEV--GAEKGLPLKVGSVFTADMFYNDEP-DWE     180
EcPNP           SKVNRIRFKDHDFAAIADF--DMVRNAVDA--AKALGIDARVGNLFSADLFYSPDGEMFD     181
EcXP            VGLNDERFGERFFSLANAYDAEYRALLQKV--AKEEGFPLTEGVFVSYPGPNFETAAEIR     214
TtPNP_II        RGPNDERLGPRFPVMFEAYDPELIELARKV--ARRQDLHLFEGVYAWFMGPSFASRAELR     214
human_PNP       RGPNDERFGDRFPAMSDAYDRTMRQRALSTWKQMGEQRELQEGTYVMVAGPSFETVAECR     206
                   .   :  :                               *

TtPNP_I         AWARYGVLAFEMEASALFLLGRMRGVRTGAILAVSNRIG---------DPELAP--PEVL     229
GtPNP           TWARYGVLAVEMETAALYTLAAKFGRKALSVLTVSDHIL---------TGEETT--AQER     229
EcPNP           VMEKYGILGVEMEAAGIYGVAAEFGAKALTICTVSDHIR---------THEQTT--AAER     230
EcXP            MMQIIGGDVVGMSVVPEVISARHCELKVVAVSAITNMAEGLSDV--KLSHAQTLAAAELS     272
TtPNP_II        LLRELGADAIGMSTVPEVIALRHLGARVLGLSTITDMAVPEREH--HATEEEVLRVAAET     272
human_PNP       VLQKLGADAVGMSTVPEVIVARHCGLRVFGFSLITNKVIMDYESLEKANHEEVLAAGKQA     266
                     *   . *..            :.   .  :::                  .

TtPNP_I         QEGVRRMVEVALEAVLEV----   247
GtPNP           QTTFNDMIEVALETAIRVE---   248
EcPNP           QTTFNDMIKIALESVLLGDKE-   251
EcXP            KQNFINLICGFLRKIA------   288
TtPNP_II        GPVFRRYVRGILAEL-------   287
human_PNP       AQKLEQFVSILMASIPLPDKAS   288
                   .   :   :
```

Supplementary Figure 5. Sequence alignment of the PNPs investigated, illustrating the difference in key nucleobase-coordinating residue.

Stability of N7X

To assess the stability of **N7X**, samples of the isolated material were incubated in citrate/HEPES/asparagine/proline universal buffer, heated, and examined by UV spectroscopy. To this end, **N7X** (ca. 100 µM) was incubated in 5 mM universal buffer (also see above) at pH 2−12 in a total volume of 200 µL in PCR tubes. These samples were incubated at 60 °C in a PCR cycler with lid heating (lid temperature set to 70 °C) for 3 h. Afterwards, the samples were cooled to room temperature and 100 µL of each sample was pipetted into 100 µL 200 mM proline/arginine buffer pH 10 in wells of a UV-transparent 96-well plate to readjust the pH of all samples and consistently observe the monoanion of **N7X**. We then obtained UV absorption spectra of these solutions from 250 to 350 nm in steps of 1 nm and the results of this experiment are shown in Supplementary Fig. 6. This experiment showed essentially identical spectra after incubation and across all pH values, suggesting that **N7X** (like most nucleosides) is very stable and does not degrade spontaneously.

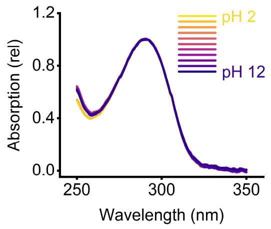

Supplementary Figure 6. UV absorption spectra of **N7X** at pH 10 after incubation at various pH values at 60 °C for 3 h.

Additional controls to exclude xanthine oxidase activity

During the revision of this manuscript, one reviewer rightfully pointed out to us that additional control experiments would be useful as nucleoside phosphorylase preparations sometimes contain relevant traces of xanthine oxidase as an impurity. Indeed, xanthine oxidase-mediated oxidation of **X** to uric acid could conceivably have caused similar redshifts in the absorption spectra as observed for the glycosylation of **X** to give **N7X**. This could have represented a relevant source of error in our kinetic measurements with *Gt*PNP (Fig. 1l). Therefore, we tested if our preparation of *Gt*PNP would (without the addition of **Rib1P**) effect a redshift of the absorption spectra of hypoxanthine or **X**, indicative of xanthine oxidase activity. To this end, we incubated 100 µM nucleobase (hypoxanthine or **X**) with *Gt*PNP (0 or 0.5 g L^{-1}) in 50 mM glycine/proline buffer pH 10 at room temperature (ca. 21 °C) in wells of a UV-transparent 96-well plate and obtained UV absorption spectra in intervals of 10 min over 1 h. These conditions reproduce the kinetic experiments and primarily observe hypoxanthine and **X** as their anions. The resulting spectra showed no indication of redshifts, or any transformation happening on the chromophore (Supplementary Fig. 7). We therefore conclude that our preparation of *Gt*PNP contained no relevant amount of xanthine oxidase. The stable equilibria observed in the glycosylation reactions (Fig. 1j and 1k) are in line with this conclusion.

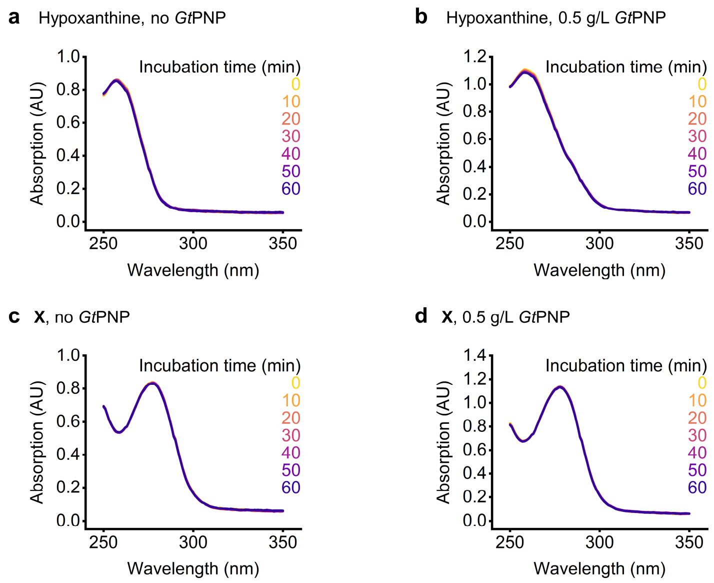

Supplementary Figure 7. Lack of oxidation of hypoxanthine (**a** and **b**) or **X** (**c** and **d**) by *Gt*PNP, confirming the absence of xanthine oxidase impurities.

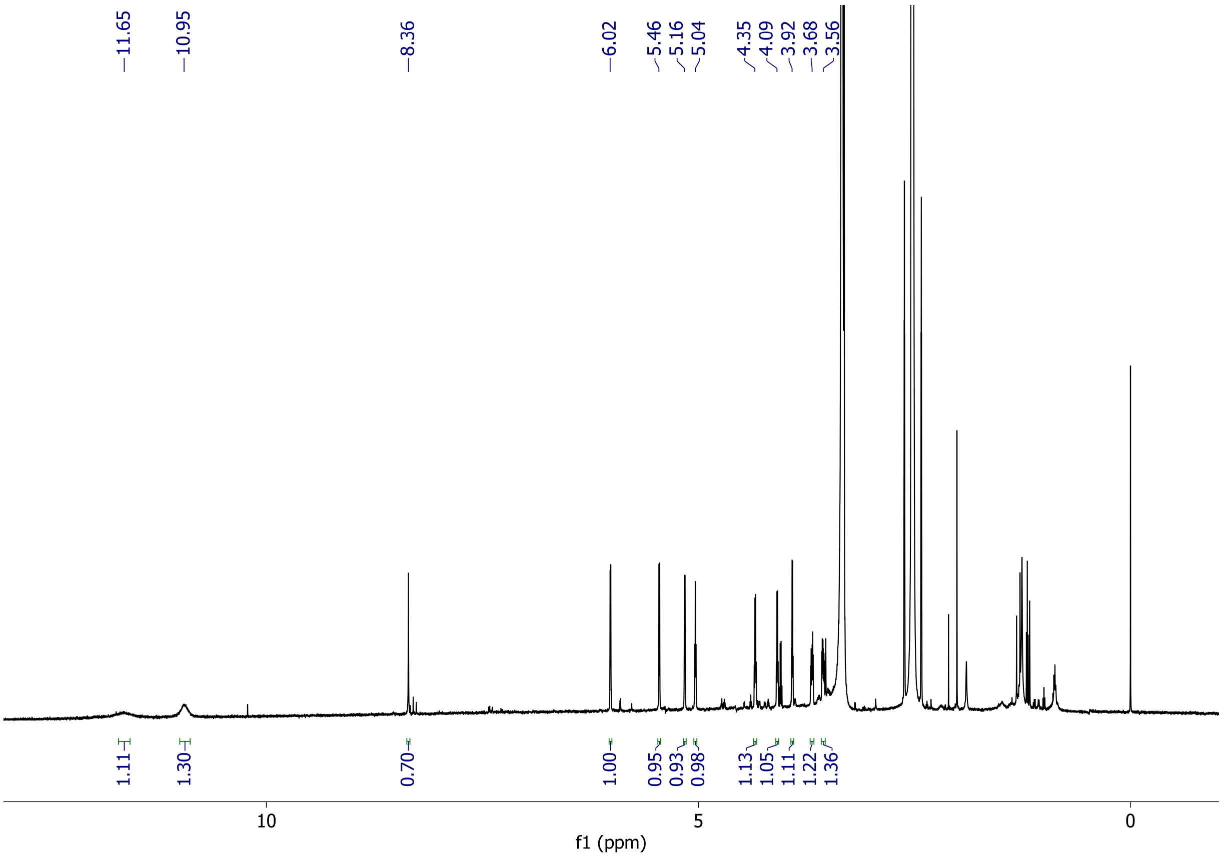

Supplementary Figure 8. ^1H NMR spectrum of **N7X** in wet DMSO-d_6. The entire spectroscopic data recorded for this project are available for download from the externally hosted supplementary information.[14]

Supplementary References

1. Zhou, X. *et al.* Recombinant purine nucleoside phosphorylases from thermophiles: preparation, properties and activity towards purine and pyrimidine nucleosides. *The FEBS Journal* **280**, 1475–1490 (2013).
2. Szeker, K. *et al.* Comparative investigations on thermostable pyrimidine nucleoside phosphorylases from Geobacillus thermoglucosidasius and Thermus thermophilus. *Journal of Molecular Catalysis B: Enzymatic* **84**, 27–34 (2012).
3. Eilert, L., Schallmey, A. & Kaspar, F. UV-Spectroscopic Detection of (Pyro-)Phosphate with the PUB Module. *Analytical Chemistry* **94**, 3432–3435 (2022).
4. Kaspar, F., Wolff, D. S., Neubauer, P., Kurreck, A. & Arcus, V. L. pH-Independent Heat Capacity Changes during Phosphorolysis Catalyzed by the Pyrimidine Nucleoside Phosphorylase from Geobacillus thermoglucosidasius. *Biochemistry* **60**, 1573–1577 (2021).
5. Kaspar, F. *et al.* Biased Borate Esterification during Nucleoside Phosphorylase-Catalyzed Reactions: Apparent Equilibrium Shifts and Kinetic Implications. *Angew. Chem. Int. Ed.* **62**, e202218492 (2023).
6. Software for Chemistry and Materials, AMS: Amsterdam Modelling Suite. http://www.scm.com-http://www.scm.com (2020).
7. Perdew, J. P., Burke, K. & Ernzerhof, M. Generalized Gradient Approximation Made Simple. *Phys. Rev. Lett.* **77**, 3865–3868 (1996).
8. Van Lenthe, E. & Baerends, E. J. Optimized Slater-type basis sets for the elements 1–118. *J. Comput. Chem.* **24**, 1142–1156 (2003).
9. Klamt, A. & Schüürmann, G. COSMO: a new approach to dielectric screening in solvents with explicit expressions for the screening energy and its gradient. *Journal of the Chemical Society, Perkin Transactions 2* 799–805 (1993) doi:10.1039/P29930000799.
10. Stephens, P. J., Devlin, F. J., Chabalowski, C. F. & Frisch, M. J. Ab Initio Calculation of Vibrational Absorption and Circular Dichroism Spectra Using Density Functional Force Fields. *The Journal of Physical Chemistry* **98**, 11623–11627 (1994).
11. Becke, A. D. Density-functional thermochemistry. III. The role of exact exchange. *The Journal of Chemical Physics* **98**, 5648–5652 (1993).
12. Lee, C., Yang, W. & Parr, R. G. Development of the Colle-Salvetti correlation-energy formula into a functional of the electron density. *Phys. Rev. B* **37**, 785–789 (1988).
13. Davidson, E. R. The iterative calculation of a few of the lowest eigenvalues and corresponding eigenvectors of large real-symmetric matrices. *Journal of Computational Physics* **17**, 87–94 (1975).
14. Kaspar, F. Supplementary Material N7X. *zenodo* (2023) doi:10.5281/zenodo.8382795.

15. Bridson, P. K., Lin, X., Melman, N., Ji, X. & Jacobson, K. A. Synthesis AND Adenosine Receptor Affinity of 7-β-D-Ribofuranosylxanthine. *Nucleosides and Nucleotides* **17**, 759–768 (1998).
16. Nicolau, C. & Hildenbrand, K. ^{13}C-Nuclear Magnetic Resonance Investigations of Xanthine and Some of its N-Methylated Derivatives. *Zeitschrift für Naturforschung C* **29**, 475–478 (1974).
17. Reijenga, J., van Hoof, A., van Loon, A. & Teunissen, B. Development of Methods for the Determination of pKa Values. *Analytical Chemistry Insights* **8**, ACI.S12304 (2013).
18. Kaspar, F. Quality Data from Messy Spectra: How Isometric Points Increase Information Content in Highly Overlapping Spectra. *ChemBioChem* **24**, e202200744 (2023).
19. Kaspar, F. *et al.* Diversification of 4'-Methylated Nucleosides by Nucleoside Phosphorylases. *ACS Catal.* **11**, 10830–10835 (2021).
20. Stachelska-Wierzchowska, A., Wierzchowski, J., Bzowska, A. & Wielgus-Kutrowska, B. Site-Selective Ribosylation of Fluorescent Nucleobase Analogs Using Purine-Nucleoside Phosphorylase as a Catalyst: Effects of Point Mutations. *Molecules* **21**, 44 (2015).
